# Parliament and the Professions

**Published:** 1920-06-26

**Authors:** 


### Parliament and the Professions.

### The Ministry of Health and Voluntary Hospitals.

Answering questions respecting the financial position of the London Hospital, the National Hospital for the Paralysed and Epileptic, and other hospitals, Dr. Addison stated that the question of giving interim assistance to hospitals in serious financial difficulties is under consideration by King Edward's Hospital Fund. The position of many hospitals gives reason for anxiety, and the Ministry of Health is considering what measures can be taken to meet the situation without prejudicing the voluntary principle and without losing the services of voluntary workers, to whose co-operation the greatest value is attached. Dr. Addison said that he had never been in favour of the nationalisation of the voluntary hospitals.

